# Under detection of depression in primary care settings in low and middle-income countries: a systematic review and meta-analysis

**DOI:** 10.1186/s13643-022-01893-9

**Published:** 2022-02-05

**Authors:** Abebaw Fekadu, Mekdes Demissie, Rahel Birhane, Girmay Medhin, Tesera Bitew, Maji Hailemariam, Abebaw Minaye, Kassahun Habtamu, Barkot Milkias, Inge Petersen, Vikram Patel, Anthony J. Cleare, Rosie Mayston, Graham Thornicroft, Atalay Alem, Charlotte Hanlon, Martin Prince

**Affiliations:** 1 Centre for Innovative Drug Development and Therapeutic Trials for Africa (CDT-Africa), Addis Ababa University, Addis Ababa, Ethiopia; 2 Department of Psychiatry, School of Medicine, College of Health Sciences, Addis Ababa University, Addis Ababa, Ethiopia; 3 Department of Global Health & Infection, Brighton and Sussex Medical School, Brighton, UK; 4 Center for Affective Disorders, Institute of Psychiatry, Psychology and Neuroscience, Department of Psychological Medicine, King’s College London, London, UK; 5 Aklilu Lemma Institute of Pathobiology, Addis Ababa University, Addis Ababa, Ethiopia; 6 Debremarkos University, Debremarkos, Ethiopia; 7 Division of Public Health, College of Human Medicine, Michigan State University, Flint, MI USA; 8 School of Psychology, College of Education and Behavioral Studies, Addis Ababa University, Addis Ababa, Ethiopia; 9 Centre for Rural Health, School of Nursing and Public Health, University of KwaZulu-Natal, Durban, South Africa; 10 Department of Global Health and Social Medicine, Harvard Medical School, Cambridge, MA USA; 11 King’s Global Health Institute, King’s College London, NE Wing Bush House, 30 Aldwych, London, WC2B 4BJ UK; 12 Centre for Global Mental Health, King’s College London, De Crespigny Park, London, SE5 8AF UK; 13 Centre for Implementation Science, Health Service and Population Research Department, Institute of Psychiatry, Psychology and Neuroscience, King’s College London, De Crespigny Park, London, SE5 8AF UK

**Keywords:** Depression, Detection, Primary health care, Review, Low and middle-income countries

## Abstract

**Background:**

Depression is one of the commonest mental disorders in primary care but is poorly identified. The objective of this review was to determine the level of detection of depression by primary care clinicians and its determinants in studies from low- to middle-income countries (LMICs).

**Methods:**

A systematic review and meta-analysis was conducted using PubMed, PsycINFO, MEDLINE, EMBASE, LILAC, and AJOL with no restriction of year of publication. Risk of bias within studies was evaluated with the Effective Public Health Practice Project (EPHPP). “Gold standard” diagnosis for the purposes of this review was based on the 9-item Patient Health Questionnaire (PHQ-9; cutoff scores of 5 and 10), other standard questionnaires and interview scales or expert diagnosis. Meta-analysis was conducted excluding studies on special populations. Analyses of pooled data were stratified by diagnostic approaches.

**Results:**

A total of 3159 non-duplicate publications were screened. Nine publications, 2 multi-country studies, and 7 single-country studies, making 12 country-level reports, were included. Overall methodological quality of the studies was good. Depression detection was 0.0% in four of the twelve reports and < 12% in another five. PHQ-9 was the main tool used: the pooled detection in two reports that used PHQ-9 at a cutoff point of 5 (combined sample size = 1426) was 3.9% (95% CI = 2.3%, 5.5%); in four reports that used PHQ-9 cutoff score of 10 (combined sample size = 5481), the pooled detection was 7.0% (95% CI = 3.9%, 10.2%). Severity of depression and suicidality were significantly associated with detection.

**Conclusions:**

While the use of screening tools is an important limitation, the extremely low detection of depression by primary care clinicians poses a serious threat to scaling up mental healthcare in LMICs. Interventions to improve detection should be prioritized.

**Systematic review registration:**

PROSPERO CRD42016039704.

**Supplementary Information:**

The online version contains supplementary material available at 10.1186/s13643-022-01893-9.

## Background

Depression is a major public health problem associated with impaired quality of life, disability and substantial healthcare costs [1]. It is a relatively common condition in primary health care (PHC), affecting up to 20% of attendees [2, 3] and adding to the burden on the healthcare system [4]. Treatment of depression leads to improvement in functioning and reduction in healthcare expenses [5]. Longer duration of untreated illness negatively influences the course and outcome of depression [6–8]; however, more than 50% of potential cases of depression remain undetected in high income countries [2]. Among those whose depression is detected successfully and who are initiated on treatment, the majority have unstructured and inadequate access to treatment [9]. The focus of primary care clinicians on ensuring that their overall decision making is right rather than on diagnosis may be partly the reason for the low detection and the type of care provided [10]. However, the belief that depression and other mental disorders are the responsibility of the specialist, the lack of the right tools, such as diagnostic and treatment guidelines, the low level of confidence to deal with depression, the clinical environment, and user level barriers are also critical barriers to the detection and treatment of depression [11].

Due to the high prevalence and the significant level of disability attributable to depression, prioritizing the detection and management of depression and taking a public health approach is critical for multiple reasons [12].

(i) Primary health care is the first entry point into the healthcare system and offers the best opportunity for detection of illness and initiation of care [13, 14]. The large treatment gap and the global effort to improve access to treatment for mental disorders [15, 16], necessitates making appropriate use of the PHC system to address the treatment gap. The recognition of depression is, therefore, an important first step in the pathway to care. However, the fact that nearly half of those with recognized depression do not receive adequate care even in high-income countries suggests a major missed opportunity to address the population-level burden of depression, including prevention of suicide. Therefore, improving detection also needs to be combined with improving capacity to provide care.

(ii) patients generally prefer to be treated for depression in primary care whenever they can [17]; (iii) the primary care facility provides service users an accessible and relatively affordable opportunity for the receipt of healthcare for neglected health problems, including depression [13]; (iv) due to the predominant presentation with somatic symptoms, most people with depression visit primary care frequently [18];

Given the overall importance of depression and its detection in PHC, this systematic review aimed to synthesize evidence about the detection of depression by PHC clinicians in LMICs, including factors that may facilitate or hinder detection. Additionally, the review aimed to evaluate the pooled prevalence of depression among the studies included for the detection of depression.

## Methods

### Search strategy

The review protocol was registered in the PROSPERO database (CRD42016039704). MEDLINE, PsycINFO, EMBASE, and PubMed databases were searched since the inception of the respective databases until 3rd week of December 2020. Latin America and Caribbean Center on Health Science Literature (LILAC) and African Journal of Online (AJOL) databases and manual search were also employed. The following terms were used to identify Depression: Depression OR depressive disorder OR Common mental disorder. The search terms used for detection were: Detection OR Detection rate OR Prevalence OR Screening OR Case finding OR Diagnosis OR Undiagnosed OR under-detection. For primary health care, we used Primary health care OR primary care OR Health centers. We used the World Bank definition and List of countries to identify LMICs. Terms for detection, depression, PHC and LMICs were combined with the Boolean term “AND” (Supplementary file 1).

### Outcomes of interest

The primary outcome was detection, defined as the proportion of the number of patients correctly diagnosed as having depression by primary care clinicians compared to a “gold” standard diagnosis. The gold standard assessment included locally validated instrument or a confirmatory clinical diagnosis by a mental health expert. The secondary outcome was prevalence of depression among studies that reported detection. Factors associated with detection were also explored.

### Eligibility criteria

Eligible articles were assessed against the following inclusion criteria.

1. Diagnosis: both adults and adolescents aged 15 years and above with depression, including major depressive disorder, bipolar depression, masked depression, secondary depression, minor depression, and sub-threshold depression as determined by primary care clinicians irrespective of the offer of intervention.
2. Study setting: LMICs at the time of the publication of the study, according to the World Bank classification (https://datahelpdesk.worldbank.org/knowledgebase/articles/906519).
3. Primary health care (PHC): Participants must have been recruited from primary health care- the first element of continuing the healthcare process [19].
4. Type of study: Prospective studies, case-control studies, cross-sectional studies, and clinical trials if aim was to evaluate impact on detection.
5. Language: No language restriction.
6. Year of publication: primary studies published since the establishment of the respective databases until 3rd week of December 2020.

### Quality assessment

Risk of bias within the studies was assessed using the Effective Public Health Practice Project (EPHPP) quality assessment tool [20]. The tool consists of eight quality assessment items: selection bias, study design, control of confounders, blinding of outcome assessors, data collection methods, withdrawals and dropouts, analysis, and intervention integrity. The last criterion was not included in overall rating because, among the nine publications, we identified only one interventional study that reported detection rate. Where follow-up studies were included, only the baseline data were used. Thus, five of the eight quality items were available for the overall rating. A global rating of “weak,” “moderate,” or “strong” were made qualitatively. The global rating was rated “strong” if no weak rating were given; moderate if only one weak rating and weak if two or more “weak” ratings were made.

The quality of reporting was assessed using Strengthening the Reporting of Observational studies in Epidemiology (STROBE) checklist containing 22 items [21] as a secondary assessment tool. We rated the 22 items by two of the authors (MD and RB) independently as per the STROBE guideline: “fully reported”, “partially reported,” and “not reported”.

### Data extraction

Studies were first screened by two of the authors (MD and RB) independently, based on their titles and abstracts and any discrepancies were reconciled through discussions with a third author (AF). Excluded articles and reasons for exclusion were documented. Data were also extracted independently by the same two authors using a piloted data extraction form that included study country, study design, sample size, number of patients detected by clinicians, number of patients detected by the “gold” standard tool, and the outcomes (detection, prevalence, associated factors).

### Statistical analysis

We conducted meta-analysis stratified by diagnostic approaches: diagnostic instrument (Beck Depression Inventory (BDI), Edinburgh Postnatal Depression Scale (EPDS); Patient Health Questionnaire-9 (PHQ-9) with the two diagnostic thresholds; Structured Clinical Interview for DSM-IV (SCID) and the Composite International Diagnostic Interview (CIDI)). Pooled prevalence estimates were obtained from two studies for PHQ-9 with the cutoff 5, from 6 studies for PHQ-9 with the cutoff-10, and from two studies for SCID based diagnosis. In the remaining two studies, depression was assessed among diabetes  clinic attendees using BDI [22] and among Antenatal Clinic (ANC) attendees using EPDS [23]. These were excluded from the meta-analysis as they were special populations, and the prevalence or detection of depression could be different. Thus, we reported these as individual studies. Furthermore, we generated two different estimates [ES] that are plotted in Figs. 2 and 3. These estimates were (a) detection of depression which was defined as the proportion of the number of patients correctly diagnosed as having depression by PHC workers compared to a “gold” standard diagnosis; (b) prevalence of depression which is the proportion of total study participants who scored above the cut-off value in the “gold” standard diagnosis used to define depression in a particular paper. Since the prevalence of depression and its detection by PHC workers is expected to be affected by several factors across different settings, heterogeneity is expected. As the pooled estimate is the average estimate of the distribution, we pooled these estimates in a random effects meta-analysis [24]. The assumptions of random effects model were tested using normal probability plot of residuals. These were approximately linear for both prevalence and detection estimates indicating that the assumptions of random effects were adequate [25]. We used the metaprop command in STATA/SE for Windows version 16 to conduct meta-analysis of detection and prevalence of depression reported in the various studies. We also assessed the normal probably plot of the residuals, which was approximately linear, supporting the condition that the error terms are normally distributed, i.e., the requirements for running a random effects model were fulfilled [25].

## Results

**Fig. 1:**
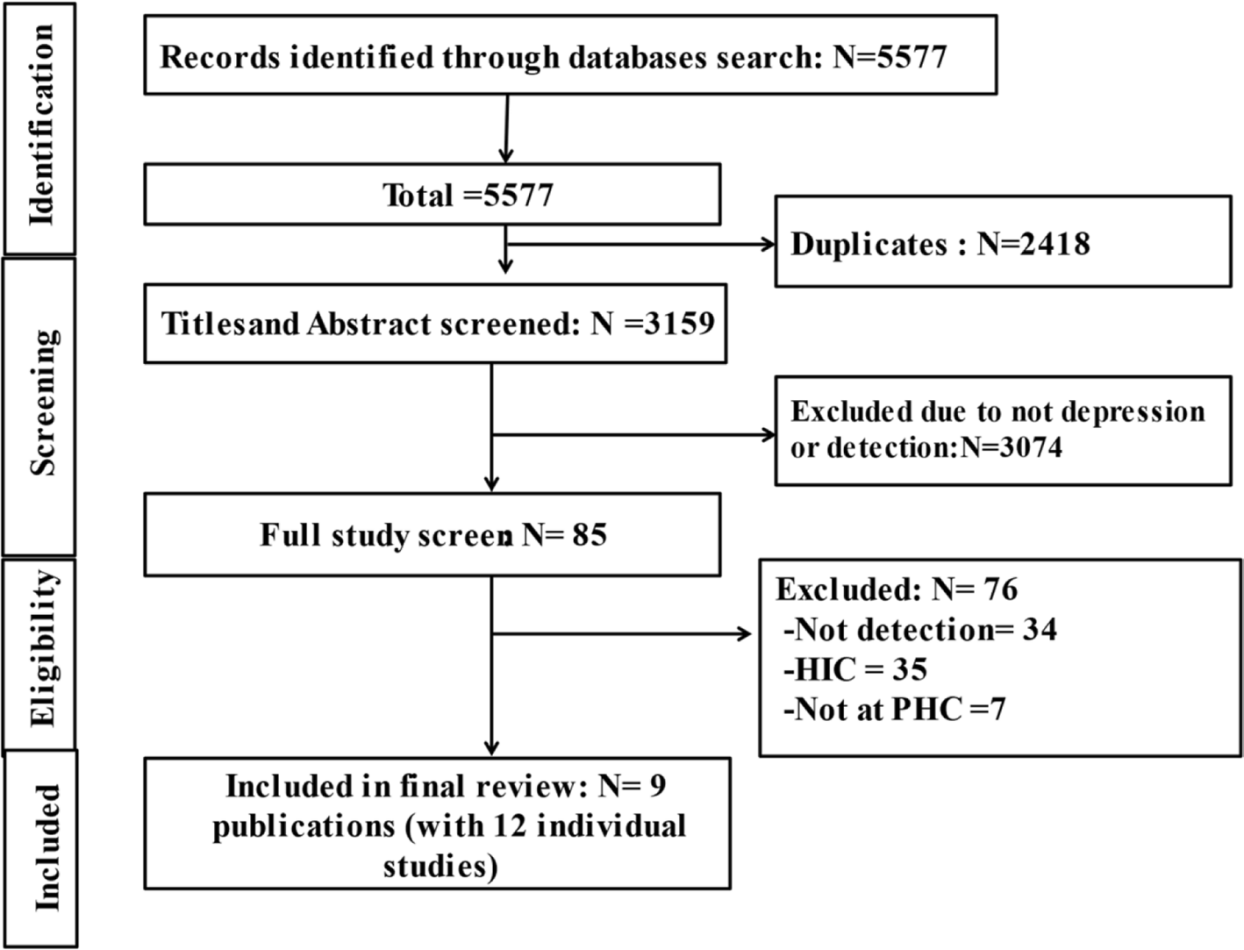
PRISMA Flow diagram of the study selection process

A total of 5577 articles were identified. After removing 2418 duplicates, 3159 titles and abstract were reviewed, and 3074 articles were excluded. A total of 85 articles were included in full article review and 9 publications with 12 country-level reports were included in the final review (Fig. 1).

### Characteristics of the included studies and case identification

The 9 publications making the 12 individual country level reports came from 2 multi-country and 7 single-country studies [26, 27]. However, we extracted only one country level report (Turkey) from a  World Health Organization (WHO) multi-country study [26] that fulfilled the inclusion criteria. The other country level reports in this study were not considered because they were not conducted in LMICs or at PHC setting (Table 1). The reports were published between 1995 and 2018 and had a total of 12,984 participants. Two reports originated each from India, Nigeria, and Malawi, and one report each from Ethiopia, Nepal, Palestine, South Africa, Turkey, and Uganda. All the studies except two were cross-sectional. The exceptions were one follow-up study [26] and one cluster randomized controlled trial [30]. For these studies, only the baseline data were included.

**Table 1:** Key characteristics of the include studies

Authors and year	Country	Setting	Study design	Sample size	Age (years)	Measurement of depression
**Single-country studies**						
**Fekadu et al. 2017** [28]	Ethiopia	PHC	Cross sectional	1014	> 18	PHQ-9 > 5 and PHQ-9 > 10
**Sweileh et al. 2014** [22]	Palestine	PHC, diabetic clinic	Cross sectional	294	> 18	BDI ≥ 16
**Udedi et al. 2014** [29]	Malawi	PHC	Cross sectional	350	> 18	SCID
**Kauye et al. 2014** [30]	Malawi	PHC	Cluster RCT	837	> 16	SCID
**Ogunsemi et al. 2010** [31]	Nigeria	PHC	Cross sectional	412	18–90	PHQ-9 > 5
**Ayinde et al. 2018** [23]	Nigeria	PHC	Cross sectional	2986	*M* = 25 (sd = 6.2)	EPDS ≥ 10
**Pal et al. 2018** [32]	India	PHC	Cross sectional	335	18–65	Psychiatrist
**Multi-country studies**						
**Rathod et al. 2018** [27]	India	CHC	Cross sectional	760	> 18	PHQ-9 > 10
	Nepal	HP and PHC	Cross sectional	1474	>16	PHQ-9 > 10
	South Africa	CHC and PHC	Cross sectional	1322	> 18	PHQ-9 > 10
	Uganda	PHC and district hospital	Cross sectional	1893	> 18	PHQ-9 > 10
**Üstün, 1995** [26]	Turkey	PHC	Cohort	1307	15–65	CIDI

*Abbreviations*: *BDI* Beck Depression Inventory, *CHC* Community Health Centre, *CIDI* Composite International Diagnostic Interview, *EPDS* Edinburgh Postnatal Depression Scale, *HPt* Health Post, *PHQ-9* Patient Health Questionnaire-9, *RCT* randomized clinical trial, *SCID* Structured Clinical Interview for DSM-IV, *M* mean, *SD* standard deviation

All studies except two were based in primary care clinics [26, 27, 29–31]. One of the remaining two studies was carried out in a primary care clinic for patients with diabetes [22] and the other in a primary maternal care clinic [23]. Moreover, one study recruited men only participants [32].

The professional background of the PHC clinicians varied across the studies depending on the health care structure and resources of each country. Thus primary care professionals included health officers [28] with a medical training of 4 years, medical officers, PHC doctors, nurses, health assistants and auxiliary health workers [26, 27]. Four studies did not report the background of the PHC clinicians [22, 29–31]. Regarding mental health training, four studies (two cross-country studies and two additional individual studies) confirm that they did not provide additional mental health training or other intervention prior to the assessment [26–28, 30] while the remaining five studies do not provide such information [22, 23, 29, 31, 32].

Clinician diagnosis of depression was recorded in a clinician consultation or encounter form [26–28] or patients’ records were reviewed and diagnosis status extracted [23, 29, 31, 32]. Two reports did not indicate the methods they used to specify clinicians’ diagnosis [22, 30].

Four studies used two stage diagnostic screening to confirm the presence of depression [26, 29, 30, 32]. In two of these studies [29, 30], participants were first screened using the 20 item Self-Reporting Questionnaire (SRQ-20) and those who scored positive on the SRQ had a confirmatory diagnostic assessment using the SCID. The other report taken from a WHO multi-country study, used the 12-item General Health Questionnaire (GHQ) for initial screening followed by confirmatory diagnosis with the primary care version of the CIDI [26]. Study participants in the second stage were all of the high scorers, 35% of the medium scorers and 10% of the low scorers. The fourth study used PHQ-9 followed by a psychiatrist assessment for confirmation of depression [32].

The remaining studies relied on a single assessment with the PHQ-9 [27, 31], the BDI-II [22] or the EPDS [23] for the diagnosis of probable depression. For the PHQ-9, varied cutoff scores were used as threshold for detection: a cutoff of threshold score of 10 [27] and score of 5 [31] and both scores of 5 and 10 [28]. One study also computed DSM-5 based diagnosis of Major Depressive Disorder (MDD) from the PHQ-9 [28].

Nine out of the 12 reports noted the order of assessment in relation to diagnosis by primary care clinician. In the reports that relied on a two-stage diagnosis (*n* = 2), the initial screening instrument was administered before clinician assessment [29, 30]. For the reports that used a single-stage screening (*n* = 7): three studies offered screening before the participants had clinician assessment, but the clinicians were blind to the results of the screening [27]; in the other four reports screening happened after assessment by clinician [23, 27, 28, 31].

### Quality of included studies

According to the EPHPP quality assessment, three studies were assessed to be of strong quality, five of moderate quality and only one study of weak quality (Supplementary file 2). The overall quality of reporting of the studies was also moderate to high as per the STROBE checklist (Supplementary file 3).

## Detection by primary care clinicians

The detection rate was 0% in four of the 12 reports [22, 27, 29, 30] that  used PHQ-9 [27], SCID [29, 30] and BDI [22] to measure depression.

Among the remaining eight studies that reported detection rate greater than 0%, the WHO study by Üstün et al (1995) used enriched sample in which participants were first screened for depression before being evaluated by primary care clinicians, whose diagnosis was compared with CIDI based ICD-10 diagnosis [26]. In this study, the detection of depression and dysthymia was 28.4% and 8.5% respectively. The study conducted by Pal et al. (2018) included only male participants and reported 69.0% detection of depression with just two physician assessors compared against psychiatrist diagnosis [32]. The third study carried out among pregnant women attending ANC follow-up reported a detection of 1.4% (*n* = 3/218) employing EPDS [23].

The pooled detection level from the two reports that used PHQ-9 with a cutoff score of 5 as a gold standard was 3.9% (95% CI = 2.3%, 5.5%). For the four reports that used PHQ-9 at a cutoff score of 10 as a “gold standard”, the pooled detection level was 7.0 % (95% CI = 3.9%, 10.2%), with no significant heterogeneity (*I*^2^ = 43.5%, *P* = 0.15).

**Fig. 2:**
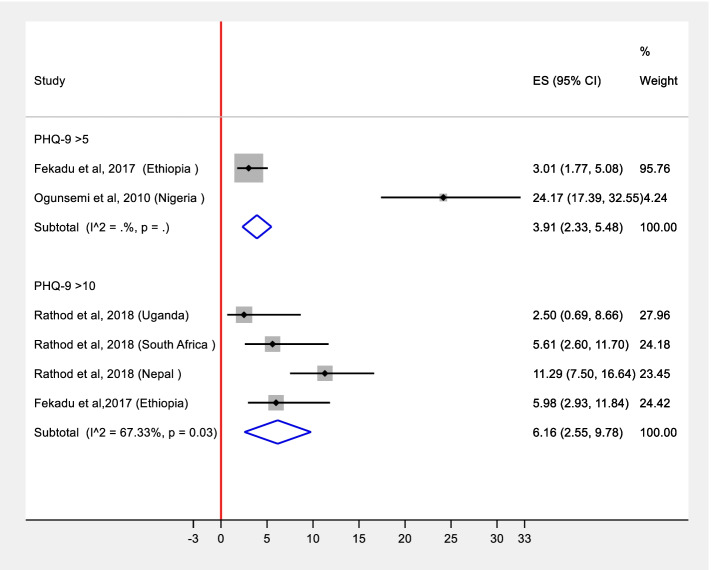
Forest plot of detecting depression by primary health care clinicians with pooled estimate stratified by the cut-off of PHQ-9 [ES = estimate of detection in percent; estimates from individual studies are pooled using random effects model]

Only one study evaluated factors associated with detection and reported severity of depression and suicidality to be associated with detection. Additionally, although not significant, women and those with higher educational attainment were more likely to be diagnosed with depression [28] (Fig. 2).

Only two out of the 12 reports presented false positivity rate [28, 32]. In the first study reported by Fekadu et al. (2017), the false positivity rate was 0.7% when the clinician identified cases were compared against PHQ-9 at a cut-off score of 10 [28]. The false positivity rate in the second report by Pal et al. (2018) was 10.9% (32/293) compared against diagnosis by psychiatrist [32].

### Prevalence of depression in the included studies

In general, the prevalence of depression was assessed using CIDI based ICD-10 diagnosis [26], EPDS [23], BDI [22], SCID [29, 30], and PHQ-9 with the cutoff score of 5 [28, 31] and 10 [27, 28].  In a study that used CIDI based ICD-10 diagnosis, the prevalence of depression and dysthymia was 11.6% and 0.9% respectively [26]. Two studies were conducted in a special participant group: the first study  was conducted among people with diabetics [22] and the second was among pregnant women attending ANC [23]. We did not include estimates from these two studies in the pooled estimate generated from the general PHC attendees. The reported prevalence figures were 40.8 % (95% CI 35.4%, 46.5%) among people with diabetes [22] and 7.3% among pregnant women who were on ANC follow-up [23].

**Fig. 3:**
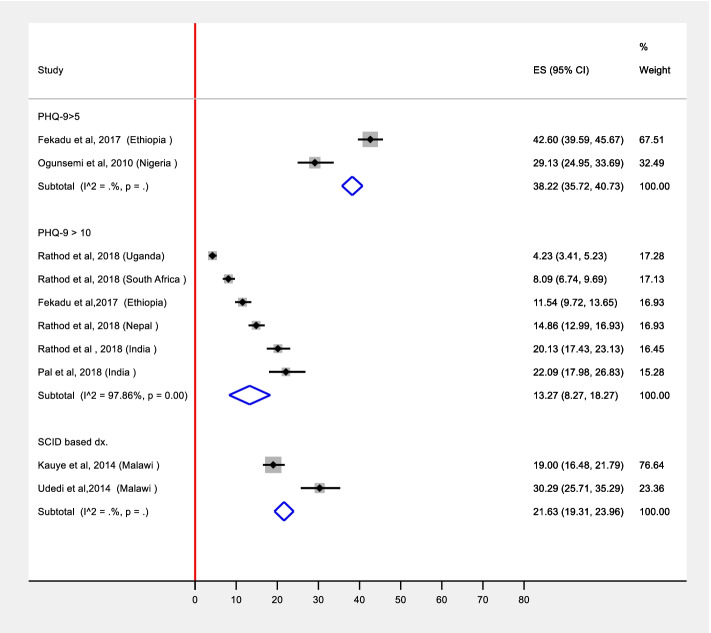
Pooled prevalence of depression stratified by diagnostic approaches using random effects model

In two studies that used SCID [29, 30], the pooled prevalence of depression was 21.6% (19.3%, 23.9%). The pooled prevalence of depression from the 12 included studies are presented in Fig. 3. In studies that used PHQ-9 at a cutoff score of 10, the pooled prevalence was 13.2 % (95% CI 8.2%, 18.2%) and it was 38.2% (95% CI 35.7%, 40.7%) among studies that used a score of 5 as a cutoff.

## Discussion

This is the first systematic review and meta-analysis of studies reporting on the detection of depression by primary care clinicians in LMICs. Overall, we identified very limited number of studies reporting on the subject. Where studies existed, the level of detection was low, with 0.0% detection in four reports [22, 27, 29, 30] and under 10% in another four reports [27, 28]. Detection was better in one of the three reports [26, 29, 30] that used enriched sample where participants were first screened for depression before being evaluated by primary care clinicians. Even in this study, the detection was 28% [26] with 0.0% in the other two studies [29, 30]. This implies that employing an initial screening has the potential for improving detection. However, screening on its own is unlikely to be sufficient.

This level of detection compares unfavourably against studies from high income countries [2], where average detection level was around 50%. The low detection cannot be explained by a low prevalence. The pooled prevalence of possible depression in this review suggests that up to a third of primary care attendees may have depression, which is consistent with the broader literature [26]. In fact, a recent systematic review reports higher prevalence of depression and depressive symptoms among outpatients in developing countries than outpatients from developed countries [33]. The low detection is a reflection of the broader neglect of people with depression in LMICs. For example, a recent global report on adequacy of treatment for depression showed that only about 4% of people with depression receive minimally adequate treatment in LMICs compared with 20% in non-LMICs [34].

In the context of the need to scale up mental health services in LMICs and reduce the treatment gap [35], this low detection rate should be of major concern. Moreover, the highprevalence and the low detection rate, means that most people with mental health problems who would benefit from treatment in primary care are not benefiting [34].

Generally, depression is under-detected and undertreated in primary care globally, even in high income countries [2, 36]. However, health system factors, such as longer engagement in care, offer a better prospect for detection in high income countries [37]. Other factors that are likely to be relevant explanations for the under-detection of depression include low prioritization, under-reporting by patients, stigma, and level of training of primary care staff. Although the use of screening instruments, such as the PHQ, may overestimate the prevalence of depression and underestimate the detection rate, it cannot explain such extremely low detection rate. Although improving the detection of depresssion should be one of the research priorities, and there is no robust evidence in LMICs on how best to improve detection. The only study indicating benefit of an intervention, a training-based approach conducted in sub-Saharan Africa [30], has not been replicated. Moreover, a similar intervention in Kenya did not improve the rate of detection [38]. Five of the studies included in this review were part of a lager study that aimed at developing the best evidence on integrating mental healthcare, including depression care, into primary care using the latest WHO guideline [35]. In this regard, a novel and complex approach is required to enhance detection and engagement in care. However, it is worth noting that a major ethical dilemma in resource limited settings is the limited availability of the needed resources to provide the required treatments. While patients identified with depression in primary care could be referred to specialist care, if available, there is no evidence to suggest that where attempts to improve detection of depression are being made that the required care is not being availed.

Only one of the included studies reported on predictors of detection, which noted severity of symptoms and suicidality as significant predictors [28]. Overall, these are consistent findings with what is known about the detection of depression [39]. Thus, the main challenge remains improving the threshold for detection, although studies on determinants of detection may be informative.

However, the review has several limitations. First, there were only few studies available for this review. We also did not search for grey literature as we believed it would be difficult to find unpublished studies of good quality. Second, there was high heterogeneity. Third, screening tools were used as gold standards, and in the case of the PHQ9, with two cutoff scores. This is likely to inflate the prevalence of depression through increasing false positives. It is also likely to underestimate the detection rate. We also focused on depression and not broader psychiatric morbidity or common mental disorders, which may be the overriding presentation in PHC. The limitations of the binary approach in the discourse and research around depression and mental disorders is well recognized [40].

## Conclusion

To our knowledge, this review is the first attempt to bring together all relevant studies on the detection of depression in primary care in LMICs. The review shows the dearth of studies on detection of depression in primary care and also highlights the challenges of improving detection. Improving detection of depression should be an important next step in scaling up services in LMICs. Depression is considered rightly a ‘priority development challenge’ [40] and the low rate of depression detection is an important barrier to addressing this development challenge. Interventions that improve detection rate by primary care clinicians in LMICs and studies on factors associated with detection are warranted. Qualitative studies should also explore the underlying reasons for the low detection beyond the obvious issues of knowledge and skills. Such studies will also inform intervention development.

## Supplementary Information

**Additional file 1.** Search strategy for systematic review of detection of depression.

**Additional file 2.** Overall quality of the studies using EPHPP.

**Additional file 3.** Assessment-based STROBE checklist.

**Additional file 4.** Standardized normal probability plot for detection.

## Data Availability

Not applicable. This is a systematic review of published literature.

## Abbreviations

BDI: Beck Depression Inventory
CHC: Community Health Centre
CIDI: Composite International Diagnostic Interview
CIDI: Composite International Diagnostic Interview
DSM-IV: Diagnostic and statistical manual of Mental Disorders, fourth edition
EPDS: Edinburgh Postnatal Depression Scale
EPHPP: Effective Public Health Practice Project
GHQ: General Health Questionnaire
LMICs: Low- and middle-income countries
mhGAP: Mental health Gap Action Program
PHC: Primary health care
PHQ-9: Patient Health Questionnaire -9
RCT: Randomized Clinical Trial
SCID: Structured Clinical Interview for DSM-IV
SRQ-20: Self-Reporting Questionnaire
STROBE: Strengthening the Reporting of Observational studies in Epidemiology
WHO: World Health Organization
