# Supplementary material for: Under detection of depression in primary care settings in low and middle-income countries: a systematic review and meta-analysis
Source: Syst Rev. 2022 Feb 5;11:21. doi: 10.1186/s13643-022-01893-9 (PMC8818168; doi:10.1186/s13643-022-01893-9)
Supplement: Supplementary file 1 — Additional file 1. Search strategy for systematic review of detection of depression. [file 13643_2022_1893_MOESM1_ESM.docx]

**Supplementary file 1: Search strategy for systematic review of detection of depression**

| Big terms |  | |
| --- | --- | --- |
| **Big term 1:**  Detection | Free term | Detection or screening or case finding or diagnosis or prevalence or undiagnos* or under-detect* or unrecognized |
|  | MeSH terms  (MEDLINE) | exp diagnosis/ or clinical decision-making/ or *signal detection, psychological/ or *symptom assessment/ or *early diagnosis/ |
|  | Emtree terms  (Embase) | exp case finding/ or exp recognition/ |
|  | Psych-INFO terms | exp Diagnosis / or exp Screening Tests/ or exp "Signal Detection (Perception)"/ |
| **Big term 2:**  Depression | Free terms | Depres* OR Major depressive disorder OR depressive symptom OR depressive state OR depressive disorder OR depression disorder OR distress OR Dysthymia OR Common mental disorder |
|  | MeSH terms  (MEDLINE) | "Depression"[Mesh] OR "Depressive Disorder"[Mesh] OR "Depressive Disorder, Major"[Mesh] OR “Dysthymic disorder” [Mesh] |
|  | Emtree terms | exp dysthymia/ or exp major depression/ or exp depression/ |
|  | Psych-INFO free terms | Depression or dysthymi$ or (depressi$ adj3 disorder$) or (depressi$ adj3 symptom$) |
|  | Psych-INFO terms | exp Postpartum Depression/ or exp Atypical Depression/ or exp Major Depression/ or exp Recurrent Depression/ or exp Late Life Depression/ or exp Dysthymic Disorder/ |
| **Big term 3:**  Primary health care | Free terms | Primary health care OR Primary care OR Health centers OR primary hospitals |
|  | MeSH terms  (Medline) | exp Primary Health Care/ or exp "Delivery of Health Care"/ or exp Community Health Centers/ |
|  | Emtree terms | exp health center/ or exp primary health care/ |
|  | Psych-INFO terms | exp Community Mental Health Services/ or exp Primary Health Care/ or exp Clinics/ |
| **Big term 4: low and middle-income countries (LMICs)** |  | exp Developing Countries/ OR ((developing OR less* developed OR third world OR under developed OR middle income OR low income OR underserved OR under served OR deprived OR poor*) adj1 (count* OR nation? OR state? OR population?)).tw OR (lmic OR lmics).tw OR exp Africa/ OR exp Asia/ OR exp South America/ OR exp Latin America/ OR exp Central America/ OR (Africa OR Asia OR South America OR Latin America OR Central America).tw OR (Afghanistan* OR Albania* OR Algeria* OR Samoa* OR Angola* OR Armenia* OR Azerbaijan* OR Bangladesh* OR Bengali OR Belarus* OR Belize OR Benin OR Bhutan* OR Bolivia* OR Bosnia* OR Herzegovina* OR Botswana* OR Brazil* OR Bulgaria* OR Burkina Faso OR Burundi* OR Cabo Verd* OR Cape Verd* OR Cambodia* OR Cameroon* OR Central African* OR Chad* OR China OR Chinese OR Colombia* OR Comoros OR Congo OR Costa Rica* OR Cote d'Ivoire OR Ivory Coast OR Cuba OR Cuban OR Djibouti OR Dominica* OR Ecuador OR Egypt OR El Salvador* OR Eritrea* OR Ethiopia* OR Fiji* OR Gabon* OR Gambia* OR Georgia* OR Ghana* OR Grenada* OR Guatemala* OR Guinea* OR Guyan* OR Haiti* OR Hondura* OR India OR Indian* OR Indonesia* OR Iran* OR Iraq* OR Jamaica* OR Jordan OR Kazakh* OR Kenya* OR Kiribati OR People* Republic of Korea OR North Korea OR Kosovo OR Kosovar* OR Kyrgyz* OR Lao OR Laos OR Laotian* OR Lebanon OR Lebanes* OR Lesotho OR Liberia* OR Libya* OR Macedonia* OR Madagascar* OR Malawi* OR Malaysia* OR Maldives OR Mali OR Malian OR Marshall Island* OR Mauritania* OR Mauriti* OR Mexico OR Mexican* OR Micronesia* OR Moldova* OR Mongolia* OR Montenegr* OR Morocc* OR Mozambique OR Myanmar OR Burmese* OR Burma OR Namibia* OR Nepal* OR Nicaragua* OR Niger* OR Pakistan* OR Palau OR Panama* OR Paraguay* OR Peru* OR Philippin* OR Romania* OR Rwanda* OR Samoa* OR Sao Tome OR Principe OR Senegal* OR Serbia* OR Sierra Leone* OR Solomon Island* OR Somalia* OR South Africa* OR Sri Lanka OR St Lucia OR Saint Lucia OR St Vincent OR Saint Vincent OR Grenadines OR Sudan* OR Suriname* OR Swaziland* OR Syria* OR Tajik* OR Tanzania* OR Thai* OR Timor* OR Togo* OR Tonga* OR Tunisia* OR Turkey OR Turkish OR Turkmen* OR Tuvalu* OR Uganda* OR Ukrain* OR Uzbeki* OR Vanuatu* OR Vietnam* OR Viet nam* OR West Bank OR Gaza* OR Palestin* OR Yemen* OR Zambia* OR Zimbabw*).tw,sh,in |
| **Final Search** | Big term 1 AND Big term 2 AND Big term 3 | |

***Abbreviation:*** MeSH, medical Subject Headings in MEDLINE
