# Supplementary material for: Under detection of depression in primary care settings in low and middle-income countries: a systematic review and meta-analysis
Source: Syst Rev. 2022 Feb 5;11:21. doi: 10.1186/s13643-022-01893-9 (PMC8818168; doi:10.1186/s13643-022-01893-9)
Supplement: Supplementary file 2 — Additional file 2. Overall quality of the studies using EPHPP. [file 13643_2022_1893_MOESM2_ESM.docx]

**Supplementary File 2: Overall quality of the studies using EPHPP**

| **Author, Year** | **Selection bias** | **Study design** | **Confounders** | **Blinding** | **Data collection methods** | **Overall Global assessment** |
| --- | --- | --- | --- | --- | --- | --- |
| Rathod et al, 2018 |  | 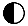 |  |  |  | Moderate |
| Pal et al, 2018 |  | 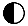 |  |  |  | Weak |
| Ayinde et al,2018 |  |  |  |  |  | Moderate |
| Fekadu et al,2017 |  | 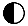 | 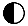 |  |  | Strong |
| Kauye et al, 2014 | 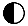 |  | NA |  |  | Strong |
| Udedi et al,2014 |  | 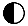 | NA |  |  | Strong |
| Sweileh et al, 2014 |  | 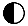 | NA |  | 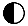 | Moderate |
| Ogunsemi et al, 2010 |  | 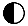 |  |  |  | Moderate |
| Üstün et al, 1995 |  | 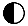 |  |  |  | Moderate |

= Strong
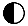
 = Moderate = weak NA= Not applicable
