# Supplementary material for: Under detection of depression in primary care settings in low and middle-income countries: a systematic review and meta-analysis
Source: Syst Rev. 2022 Feb 5;11:21. doi: 10.1186/s13643-022-01893-9 (PMC8818168; doi:10.1186/s13643-022-01893-9)
Supplement: Supplementary file 3 — Additional file 3. Assessment-based STROBE checklist. [file 13643_2022_1893_MOESM3_ESM.docx]

| **Author, Year** | Title and abstract | Background/rationale | Objectives | Study design | Setting | Participants | Variables | Data source &  measurement | Bias | Sample size | Quantitative variables | Statistical methods | Participants | Descriptive data | Outcome data | Main results | Other analysis | Key results | Limitations | Interpretations | Generalizability | Funding |
| --- | --- | --- | --- | --- | --- | --- | --- | --- | --- | --- | --- | --- | --- | --- | --- | --- | --- | --- | --- | --- | --- | --- |
| Rathod et al, 2018 |  |  |  |  | 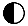 |  |  |  |  |  |  |  |  |  |  |  |  |  |  |  |  |  |
| Pal et al,2018 |  |  |  |  |  |  |  |  | 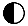 |  |  |  |  |  |  |  |  |  |  |  |  |  |
| Ayinde et al,2018 |  |  |  |  |  |  |  |  | 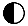 |  |  |  |  |  |  |  |  |  |  |  |  |  |
| Fekadu et al,2017 |  |  |  |  | 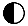 |  |  |  |  |  |  |  |  |  |  |  |  |  |  |  |  |  |
| Kauye et al, 2014 |  |  |  |  |  |  |  |  |  |  |  |  |  |  |  |  |  |  |  |  |  |  |
| Udedi et al,2014 |  |  | 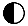 |  |  |  |  |  |  |  |  |  |  |  |  | 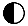 |  |  |  |  |  |  |
| Sweileh et al, 2014 |  |  |  |  |  |  |  |  |  |  |  |  |  |  |  |  |  |  |  |  |  |  |
| Ogunsemi et al, 2010 | 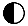 |  |  | 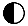 | 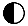 |  |  |  |  |  |  |  | 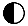 |  |  | 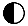 |  |  |  |  |  |  |
| Üstün et al, 1995 |  |  |  |  |  |  |  |  | 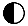 |  |  |  |  |  |  |  |  |  |  |  |  |  |

**Supplementary File 3: Assessment-based STROBE checklist**

= Fully reported
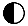
 = Partially reported = Not reported
