# Supplementary material for: Under detection of depression in primary care settings in low and middle-income countries: a systematic review and meta-analysis
Source: Syst Rev. 2022 Feb 5;11:21. doi: 10.1186/s13643-022-01893-9 (PMC8818168; doi:10.1186/s13643-022-01893-9)
Supplement: Supplementary file 4 — Additional file 4. Standardized normal probability plot for detection. [file 13643_2022_1893_MOESM4_ESM.docx]

Standardized normal probability plot for detection

Standardized normal probability plot for prevalence
